# From Feeding Difficulties to Nutritional Risk in Children with Down Syndrome

**DOI:** 10.3390/nu18193295

**Published:** 2026-10-08

**Authors:** Maja Ergović Ravančić, Valentina Obradović, Svjetlana Škrabal

**Affiliations:** Faculty of Tourism and Rural Development in Požega, Josip Juraj Strossmayer University of Osijek, Vukovarska 17, 34000 Požega, Croatia; mergovic@ftrr.hr (M.E.R.); sskrabal@ftrr.hr (S.Š.)

**Keywords:** down syndrome, trisomy 21, pediatric feeding disorder, dysphagia, breastfeeding, complementary feeding, food texture, food neophobia, dietary diversity, nutritional risk

## Abstract

Children with Down syndrome (DS) may experience feeding difficulties arising from interactions among hypotonia, cardiorespiratory and gastrointestinal conditions, swallowing dysfunction, oral-motor and sensory differences, delayed feeding skill development, and psychosocial or environmental factors. These difficulties may compromise feeding safety and efficiency, restrict dietary diversity, and contribute to inadequate fluid or nutrient intake, faltering growth, micronutrient deficiencies, or excess adiposity. This narrative review synthesized the literature selected for its relevance to feeding development and nutritional risk in children and adolescents with DS. Sources were identified through PubMed/MEDLINE, Scopus, and the Web of Science Core Collection, supplemented by reference-list review and citation tracking; cited sources were last verified on 26 August 2026. Evidence was organized according to developmental stage and clinical domain, with attention to study design, sampling, assessment methods, and applicability to the pediatric DS population. The findings indicate that breastfeeding is possible but may require early assessment of milk transfer and nutritional adequacy. Oropharyngeal dysphagia and silent aspiration are important clinical concerns, although much of the evidence comes from selected clinical and referral-based cohorts. Impaired chewing, delayed texture progression, and food selectivity may restrict the consumption of meat, vegetables, whole grains, and other nutrient-dense foods, potentially reducing dietary variety and the intake of protein, fiber, and specific micronutrients. Reliance on a narrow range of accepted soft or energy-dense foods may nevertheless provide adequate or excessive energy, allowing poor dietary quality to coexist with excess body weight. Nutritional assessment should therefore consider food choices, texture adaptations, and actual nutrient intake alongside growth and body mass index. The strongest support is for systematic, individualized, and multidisciplinary care integrating swallowing safety, feeding skills, actual dietary and fluid intake, growth, biochemical status, associated medical conditions, and family circumstances. Evidence for specific feeding therapies, supplements, and nutrition-related interventions remains limited by small samples, heterogeneous methods, and short follow-up. More representative longitudinal and intervention studies using standardized, age-appropriate outcomes are needed.

## 1. Introduction

Down syndrome (DS) results from a complete or partial additional copy of chromosome 21 [1,2]. It is associated with a highly variable combination of hypotonia, developmental delay, craniofacial and dental characteristics, congenital heart disease, respiratory disease, gastrointestinal disorders, thyroid dysfunction, and celiac disease [2,3,4,5]. These conditions and phenotypic characteristics may influence how a child obtains, processes, or tolerates food [3,4,5,6]. Feeding and nutrition problems should therefore not be attributed solely to a presumed “slow metabolism” or food preferences [5,7]. Assessment should begin with function and determine whether the child can maintain physiological stability, initiate and sustain sucking, coordinate swallowing and breathing, protect the airway, form and transport a food bolus, manage developmentally appropriate textures, respond to hunger and satiety cues, and participate in mealtimes without excessive effort or distress [3,5,6,7,8,9,10,11,12,13].

A feeding difficulty becomes clinically significant when it compromises safety, feeding efficiency, adequacy of intake, development, or participation in family mealtimes. The consensus framework for pediatric feeding disorder defines the condition as impaired oral intake that is not age-appropriate and is associated with dysfunction in one or more of four domains: medical, nutritional, feeding skill, and psychosocial [7]. This multidomain definition is particularly applicable to children with DS because it captures the interaction among medical conditions, functional abilities, nutritional status, caregiver responses, and the feeding environment [5,7,14]. For example, silent aspiration represents both a medical risk and impaired feeding function and may require modification of food or fluid consistency, feeding technique, or route of nutrition [3,9,10,11,12,13]. Although such modifications may improve safety, prolonged dependence on puréed foods after a child is developmentally ready to progress may restrict dietary variety and reduce opportunities to practice chewing and accept more complex textures [8,14,15,16].

Inadequate intake or faltering growth may heighten caregiver concern and mealtime stress [17]. Observational studies in children with DS have associated reported feeding difficulties with food refusal and negative affect during meals, while autonomy-supportive prompts have been more frequently followed by immediate food acceptance than coercive or controlling prompts [18,19,20]. These findings do not establish a simple unidirectional causal pathway, but they support assessing the interaction between the child’s feeding abilities and caregiver responses. Limited access to specialist support may further delay appropriate management [17,21], while financial and practical constraints can restrict access to varied, nutrient-dense foods and contribute to the persistence of feeding difficulties [21].

When feeding difficulties are not identified and addressed promptly, their nutritional consequences may vary with the child’s age, functional abilities, and comorbidities. During infancy, inefficient feeding can result in inadequate milk and fluid intake, dehydration, and faltering growth [3,5,9,22,23,24,25,26]. As the child develops, persistent texture restriction and selective eating may narrow the dietary repertoire and reduce the intake of fiber and nutrient-dense foods, including iron-rich foods, vegetables, fish, nuts, and whole grains [14,27,28,29,30,31]. At the same time, excess adiposity becomes an increasingly important concern during later childhood and adolescence [5,27,28,29,32,33,34,35,36,37,38]. Excess body weight does not exclude nutritional risk because a child may have excess adiposity while consuming a restricted diet or having an inadequate intake or status of specific micronutrients [5,27,28,29,39].

Body mass index (BMI) is a useful screening measure but does not directly measure adiposity or establish dietary adequacy [32,36,38]. Nutritional assessment should therefore integrate serial growth patterns, hydration, dietary intake and diversity, biochemical indicators when clinically indicated, feeding safety, and participation in feeding and family mealtimes [5,7,32,36]. Assessment of body composition may provide additional information when it is available and clinically relevant [32,36].

Previous reviews provide important foundations for understanding feeding and nutrition in DS. Nordstrøm et al. reviewed nutritional challenges across childhood and adolescence [5], Hielscher et al. examined complementary feeding and early eating experiences [14], and other reviews addressed breastfeeding and micronutrient status [22,23,39]. Building on these contributions, the present review brings swallowing safety, feeding skills, dietary exposure, nutritional status, and family circumstances into a developmental framework organized around the four domains of pediatric feeding disorder. Its contribution is the clinical integration of these domains, with particular attention to the distinction between observed associations and proposed mechanisms, and between improved feeding function and demonstrated nutritional or safety benefits. This perspective helps explain why both inadequate intake and excess adiposity need to be considered when assessing nutritional risk in children with feeding difficulties.

## 2. Review Methodology

A narrative review was undertaken to examine feeding development and nutritional risk in children and adolescents with DS. The review integrated literature on swallowing safety, feeding skills, food acceptance, caregiver practices, dietary intake, growth, micronutrient status, associated medical conditions, and nutritional management. Its purpose was to relate these clinical domains across development rather than estimate a pooled prevalence or treatment effect.

Literature was identified through PubMed/MEDLINE, Scopus, and the Web of Science Core Collection, supplemented by reference-list review and citation tracking. Search concepts included Down syndrome or trisomy 21 in combination with terms relating to breastfeeding, swallowing, dysphagia, aspiration, chewing, complementary feeding, food texture, food selectivity, food neophobia, dietary intake, growth, micronutrients, obesity, and feeding interventions. No lower publication date limit was applied. Cited sources were last verified on 26 August 2026.

Sources were selected according to the authors’ assessment of their relevance to the review’s clinical questions. Particular consideration was given to the population studied, the outcomes assessed, and the contribution of each source to understanding feeding difficulties and their potential nutritional implications. Mixed-age studies were interpreted with attention to their applicability to children and adolescents. Adult evidence was used only for context where pediatric evidence was limited.

Original studies informed the discussion of study populations, observed frequencies, associations, and intervention outcomes. Previous reviews contextualized the findings and broader evidence gaps, while guidelines and consensus statements informed clinical recommendations. General pediatric evidence was used when DS-specific evidence was limited, with its indirect applicability identified in the text.

Study design, sampling, participant characteristics, assessment methods, reported limitations, and relevance to the pediatric DS population informed the narrative interpretation. Selected study characteristics are summarized in the tables accompanying the relevant sections below.

Evidence was synthesized by developmental stage and clinical domain. Interpretation distinguished clinically referred samples from population-based samples, caregiver reports from directly measured outcomes, screening findings from instrumentally confirmed abnormalities, and cross-sectional associations from temporal or causal evidence. Related publications from the same cohort were considered together rather than as independent replications. Intervention findings were interpreted separately for feeding function, nutritional outcomes, and safety.

No meta-analysis or formal GRADE assessment was performed. Three descriptive categories were used to summarize the support for specific findings and clinical practices. A practice was considered established when supported by relevant guidelines or consistent findings with clear clinical applicability. Evidence was described as emerging or conditional when findings were preliminary or an approach required further evaluation. Evidence was considered insufficient when the available studies did not support a particular conclusion or routine clinical use. These categories represent a narrative interpretation of the literature rather than formal ratings of evidence certainty. For example, clinically indicated swallowing assessment was considered established practice because it is supported by relevant guidance and clinical findings [3,9,10,11,12,13]. However, the selected clinical samples in these studies do not support population prevalence estimates. Improvements in feeding skills reported in small intervention studies were considered emerging evidence because of limitations in sample size, study design, and outcome assessment [40,41]. Evidence from these studies was considered insufficient to establish nutritional or respiratory benefits because those outcomes were not assessed.

The relevance-based selection of literature was not intended to provide an exhaustive or fully reproducible inventory of studies. Selection and citation bias therefore remain limitations of this narrative review.

## 3. From Feeding Difficulty to Nutritional Risk: A Conceptual Framework

The main terms used in this review are defined in Table 1 to distinguish feeding difficulties from specific swallowing, feeding skill, and behavioral concerns.

These terms describe different aspects of feeding and may overlap in the same child.

**Figure 1:**
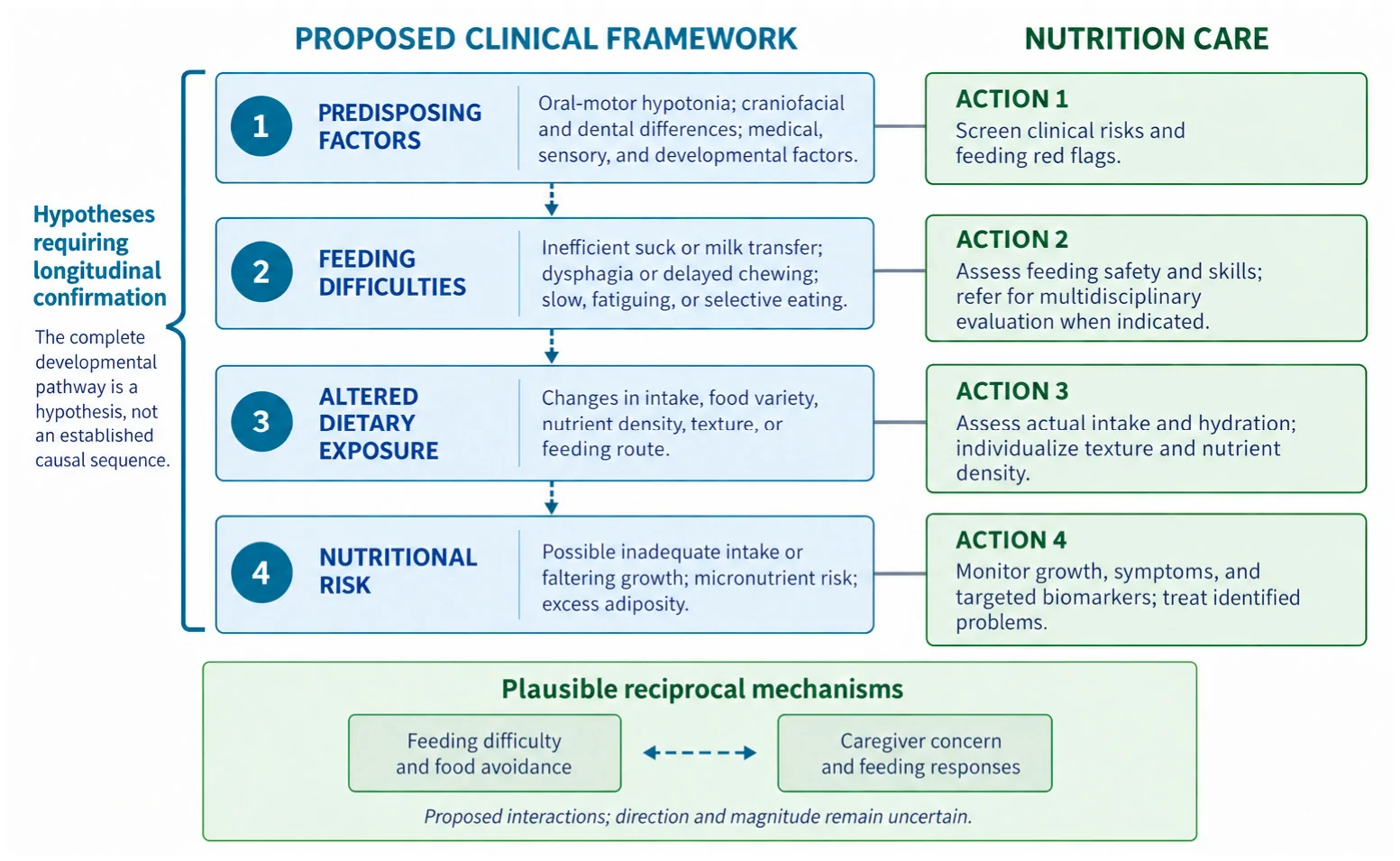
Proposed framework relating feeding difficulties to nutritional risk in children with Down syndrome (Dashed arrows indicate hypothesized relationships requiring longitudinal confirmation rather than established causal pathways. The bidirectional dashed arrow represents a proposed reciprocal relationship between feeding difficulty and food avoidance and caregiver concern and feeding responses; its direction and magnitude remain uncertain.).

The premise of this review is that feeding difficulties are not an anthropometric diagnosis but a process through which a child may be exposed to nutritional risk. Whether this process leads to an adverse nutritional outcome depends on the interaction among the four domains encompassed by the concept of pediatric feeding disorder: medical, nutritional, feeding skill, and psychosocial [7]. Medical stability influences endurance during mealtimes, appetite, and the digestion and absorption of nutrients. Feeding skills determine whether a child can consume food safely and efficiently, whereas the nutritional domain encompasses the amount, variety, and energy and nutrient density of the foods available in a form that the child can manage. Psychosocial factors determine whether mealtimes promote learning and independence or are characterized by fear, pressure, and avoidance. The feasibility of dietary recommendations is also influenced by the availability of appropriate foods, family resources and routines, and access to professional support [17,21,29,44]. Figure 1 distinguishes observed relationships, plausible reciprocal mechanisms, and hypotheses requiring longitudinal confirmation. It is an integrative framework proposed by the authors; the complete developmental pathway has not been validated as a causal model.

During infancy, feeding difficulties often begin with reduced alertness, hypotonia, respiratory instability, or congenital heart disease, which may impair milk transfer, increase fatigue, and prolong feeding [9]. Oropharyngeal dysphagia may be present even when feeding appears outwardly effective. Because aspiration in children with DS is frequently silent, the absence of coughing does not exclude impaired airway protection [10,11,12,13]. Depending on the clinical and instrumental assessment, the feeding plan may include expressed breast milk, modification of teat flow and feeding pace, thickening under specialist guidance, fortification of feeds, or temporary or longer-term enteral support [9,12,13]. Each of these strategies changes both nutrient delivery and the child’s oral feeding experience. Modifications introduced for safety should therefore be reviewed regularly as the child’s physiology and feeding skills develop. If continued unnecessarily, they may restrict progression to more complex textures and reduce opportunities for oral learning. Conversely, advancing food textures without reassessing swallowing function may increase the risk of aspiration [10,11,12,13].

During the introduction of complementary foods, delayed postural control, incomplete lip closure, limited tongue lateralization, and inefficient chewing may restrict the range of textures that the child can manage [14,45,46]. Sensory differences may impose additional constraints by affecting the child’s responses to food hardness, graininess, size, or mixed consistencies [47,48,49]. Families often make understandable adaptations by blending foods, selecting softer options, or repeatedly offering a small number of meals that the child can eat quickly and reliably [14,50]. Such adaptations may preserve food intake in the short term. However, if they become a persistent pattern, they may reduce exposure to vegetables, meat, pulses, whole grains, nuts prepared in a safe form, and mixed texture meals [14]. Unpleasant experiences associated with particular textures, such as gagging, choking, or difficulty processing food, may further reinforce the expectation of discomfort and subsequent food avoidance [47,48,49]. At the same time, caregiver concerns about choking, inadequate intake, or faltering growth may lead to more intensive prompting and the preparation of separate meals [14,17,44,50]. These interacting responses may create a feedback loop in which an initial functional limitation is followed by reduced food exposure, greater avoidance, increased caregiver concern, and an increasingly restricted dietary repertoire. This sequence should be understood as a potentially reciprocal process rather than a proven unidirectional causal pathway [7,14].

The final nutritional outcome depends on the severity and duration of the feeding difficulties and the context in which they occur [5,7]. An inadequate volume of food and fluid may lead to protein-energy malnutrition, dehydration, and faltering growth [51]. Limited dietary diversity may reduce the intake of fiber and specific micronutrients, whereas reliance on readily accepted soft, refined, or energy-dense foods may increase energy intake despite a restricted dietary repertoire [52]. Texture modification may also decrease or increase the energy density of meals, depending on the foods selected, the amount of fluid added, and the method of preparation. Initial feeding difficulties may subsequently be compounded by constipation and reflux [51,53] and by iron deficiency [50], which may further reduce appetite or feeding capacity.

## 4. Early Feeding Development

### 4.1. Breastfeeding: Potential, Barriers and Nutritional Consequences

DS is not a contraindication to breastfeeding [3,22,23,54]. Depending on the infant’s clinical condition and swallowing safety, human milk may be provided directly at the breast, as expressed milk, fortified when indicated, or in combination with other feeding routes [3,22,23,24,25,26]. Successful lactation depends on both maternal milk production and effective milk transfer by the infant, which should be assessed separately [22,23,26]. Hypotonia, reduced alertness, weak or unsustained sucking, ineffective latch, prolonged hospitalization, surgery, and cardiorespiratory fatigue may impair milk transfer even when maternal milk production is adequate [3,22,23,24,25,26]. Conversely, introducing infant formula without a plan for regular milk expression may reduce breast stimulation and compromise milk production before the infant’s feeding ability improves [54]. Assessment should therefore integrate serial changes in body weight, urine output, direct observation of latch and milk transfer, audible or visible swallowing, feeding endurance, and signs of satiety after feeding [3,22,23,54].

The 2022 systematic review by Magenis et al. [22] included 16 studies involving 2022 children with DS. The included studies differed substantially in their designs, study periods, definitions, and the children’s ages at the time of maternal recall. Estimates of exclusive breastfeeding ranged from 31.6% to 55.4%, whereas rates of breastfeeding initiation or any breastfeeding ranged from 48% to 92.5%. These ranges reflect heterogeneous studies rather than a single expected rate. A 2021 scoping review also identified barriers related to the infant, mother, and healthcare professionals and concluded that evidence on effective breastfeeding support specifically tailored to DS remains scarce [23].

In a survey conducted at a Mexican clinic among 100 mothers of children younger than five years, 98% reported that their infant had received breast milk at some point, but 64% identified infant formula as the primary source of nutrition during the first three days of life [25]. The median duration of breastfeeding was 91.5 days, and 24 of the 74 infants with complete data on exclusive breastfeeding were exclusively breastfed for six months. The single-center, recall-based design and the high prevalence of congenital heart disease limit the generalizability of these findings [25].

Qualitative findings help explain why breastfeeding rates alone are insufficient to understand the feeding experiences of children with DS. In a study involving seven Swedish mothers, participants described inconsistent professional advice, preconceived assumptions that breastfeeding would not be successful, and the importance of timely, knowledgeable, and sustained support [26]. At the same time, a Brazilian study of infants with DS and congenital heart disease showed that medical complexity may substantially limit feeding options, although such findings do not, in themselves, justify discontinuing breastfeeding [24]. Rather than treating breastfeeding and medical safety as competing priorities, lactation goals should be aligned with safe and nutritionally adequate feeding. In this context, the nutritionist’s role includes arranging early expert observation of feeding between mother and infant, supporting the maintenance of milk production during separation, and assessing whether the infant’s actual intake meets fluid, energy, and nutrient requirements. This approach both protects the infant’s health and reduces unnecessary burden on the mother: lactation success should not be presented as a measure of parental commitment, but concerns about aspiration, inadequate intake, or faltering growth should not be disregarded for the sake of maintaining exclusive breastfeeding [24,26]. Key pediatric dysphagia studies and their design limitations are compared in Table 2.

### 4.2. Sucking, Swallowing and Silent Aspiration

Oropharyngeal dysphagia is one of the most clinically consequential early feeding risks in children with DS. Hypotonia, reduced oral pressure, poor coordination of sucking, swallowing, and breathing, airway anomalies, and cardiorespiratory disease may affect both the oral and pharyngeal phases of swallowing. Overt coughing is a useful sign when present, but it is not sufficiently sensitive because aspiration is frequently silent in children with DS. Recurrent respiratory symptoms, oxygen desaturation during feeding, wet or congested breathing, poor endurance, prolonged feeding times, changes in color during feeding, an unexplained need for supplemental oxygen, and faltering growth should therefore lower the threshold for specialist assessment [3,6,9,10,11,12,13].

The swallowing studies summarized below were conducted in selected clinical or referral-based samples. Their findings describe the children assessed and should not be interpreted as population prevalence estimates for DS [9,10,11,12,13]. Stanley et al. [9] retrospectively reviewed 174 infants aged 0–6 months attending a specialized DS clinic. Clinical concerns prompted referral for videofluoroscopic swallowing study (VFSS) in 100 infants (57%). Of the entire cohort, 96 infants (55%) had oral and/or pharyngeal dysphagia, while 69 (39%) required modification of liquid consistency or nonoral feeding. Airway or respiratory abnormalities and oxygen desaturation during feeding were strongly associated with dysphagia [9]. Other cohorts of clinically referred patients demonstrate a consistent pattern. Among 127 infants who underwent VFSS, Narawane et al. [10] reported at least one oral phase abnormality in 89.8% and pharyngeal dysphagia in 72.4%. Abnormal sucking and inadequate bolus formation or control were common. Jackson et al. [11] analyzed 247 VFSS examinations and 14 fiberoptic endoscopic evaluations of swallowing in 111 infants. Deep laryngeal penetration or aspiration was identified in 31.9% of infants younger than six months and 51.3% of those aged 6–12 months. In a broader pediatric VFSS cohort of 158 children, pharyngeal dysphagia was identified in 56.3%. Among the 61 children who aspirated, 90.2% did so silently, and oral phase findings did not reliably predict pharyngeal impairment [12]. O’Neill and Richter identified VFSS-confirmed pharyngeal dysphagia in 116 of 201 children with complete records at a tertiary center, while resolution of dysphagia was documented in only 20 children during the available follow-up period [13].

A 2025 cross-sectional study of 335 children aged 4–12 years used the Functional Oral Intake Scale, the Screening Tool of Feeding Problems, and clinical observation. Overall, 25.9% of participants were classified as not receiving oral feeding, while 33.7% were considered at risk of aspiration. These are sample-specific screening findings. The reported “aspiration risk” was based on screening items and clinical observation, not instrumental confirmation of aspiration. The study may therefore support clinical triage and hypothesis generation, but it cannot replace VFSS or fiberoptic endoscopic evaluation of swallowing (FEES) when the findings would alter clinical management [57].

Following assessment, nutritional management should translate clinical and physiological findings into a safe and nutritionally adequate feeding plan [3,7,16]. Changes in teat flow rate, feeding pace or position, liquid thickness, food texture, feeding route, or meal duration may alter the volume consumed and consequently affect total water, energy, and nutrient intake. Thickening itself does not reduce intestinal water absorption when an equivalent volume is consumed. The principal nutritional concern is that altered palatability, flow, preparation, or feeding effort may reduce the volume actually consumed [58].

General dysphagia guidance indicates that the final thickness of a liquid may vary with the thickening product, liquid base, temperature, preparation method, and time elapsed after preparation [16,58,59]. The International Dysphagia Diet Standardization Initiative (IDDSI) provides a standardized framework for describing and testing liquid thickness and food texture, thereby facilitating communication among professionals and caregivers [16]. However, IDDSI does not determine which level is appropriate for an individual child; this decision requires individual clinical and, when indicated, instrumental assessment [16].

Every prescribed modification should be accompanied by a precise written recipe, practical caregiver demonstration, and verification of its compatibility with infant formula, expressed human milk, medications, and the intended feeding equipment [16,58]. The plan should specify a date or clinical trigger for reassessment and include monitoring of respiratory symptoms, actual food and fluid intake, hydration, growth, tolerance, and feeding skill development [3,7,16].

### 4.3. Complementary Feeding and Safe Texture Progression

General pediatric guidance recommends that complementary foods provide an increasing share of energy and nutrient requirements from approximately six months of age, while human milk or infant formula feeding continues [8,15]. However, decisions regarding the initiation of complementary feeding and the selection of an appropriate texture should not be based solely on chronological age [8,14,15]. A child’s readiness encompasses physiological stability, head and trunk control, the ability to bring objects to the mouth, an interest in food, and the ability to manage the offered texture safely. A diagnosis of DS alone is not a reason to postpone the introduction of food indefinitely. Conversely, reaching a particular chronological age should not take precedence over airway safety. The aim is to offer the most developmentally advanced texture that the child can manage safely and efficiently at that time, with planned progression toward more complex textures rather than prolonged reliance exclusively on smooth purées [8,14,15,16].

In their 2023 scoping review, Hielscher et al. included 18 studies involving a total of 1276 children and summarized the available evidence on complementary feeding and early eating experiences in children with DS [14]. The included studies suggested that complementary or solid foods were introduced later than in typically developing peers, that progression toward more complex textures was slower, and that motor and sensory difficulties influenced the feeding process. More cautious or controlling caregiver strategies were also observed and may be understood in the context of concerns about feeding safety and the child’s intake. The strength of these conclusions is limited by the characteristics of the available evidence. Only two intervention studies were identified; none of the studies followed children longitudinally; six samples were recruited in clinical settings, and 13 of the 18 studies were conducted in Western countries. The review therefore provides a relatively consistent account of the developmental course of these difficulties but cannot establish the optimal timing for introducing specific textures or confirm the effectiveness of any particular protocol for texture progression [14].

A subsequent survey of caregivers of 75 infants and young children with DS and 66 typically developing children found differences in complementary feeding practices and concerns. Caregivers of children with DS were also less likely to report receiving information about complementary feeding. However, the cross-sectional, caregiver-reported design does not establish causal relationships or the effectiveness of particular feeding strategies [60].

These findings highlight the need to prevent two opposing clinical risks simultaneously. The first is the premature introduction of a texture that the child cannot yet process safely by forming, controlling, and swallowing an appropriate bolus, particularly when swallowing safety has not been adequately assessed. Such progression may increase the risk of choking or aspiration [10,11,12,13,16], while repeated adverse experiences may further intensify fear and food avoidance [14]. The second risk is the unnecessary continuation of a uniform smooth texture after the child has demonstrated readiness for further learning. This may restrict opportunities to develop chewing and self-feeding skills, participate in family meals, and achieve greater dietary variety [8,14,15,45,46]. Texture advancement should therefore be planned individually and in collaboration with a feeding therapist, beginning with the child’s documented current abilities and a clearly defined next step [14,16,50]. At each safe texture level, the nutritionist should ensure that the foods provided meet the child’s requirements for energy, protein, and micronutrients while supporting the greatest possible dietary variety [8,15]. Because intervention evidence remains sparse, this approach should be regarded as individualized clinical practice rather than a validated, uniform protocol for texture progression specific to children with DS [14].

## 5. Feeding Difficulties Beyond Infancy

### 5.1. Oral Motor Function, Chewing, and Mealtime Efficiency

As children transition from liquids and puréed foods to family foods, chewing ability becomes a major determinant of dietary variety. Children with DS may have reduced jaw stability and strength, ineffective lip closure, limited tongue lateralization, differences in tooth eruption, malocclusion, and difficulty adapting masticatory movements to food hardness. These features may manifest as prolonged oral holding, pocketing of food in the cheeks, sucking or mashing rather than chewing, expulsion of food pieces, overstuffing of the mouth, fatigue, and swallowing of inadequately processed food. A parental report that a child “does not like meat” may therefore reflect the motor demands of eating it rather than a taste preference [45,46,55,56,61].

Direct measurements of masticatory function remain limited. Wintergerst and López-Morales compared eight children with DS aged 8–10 years with age-matched peers and reported a maximum occlusal force approximately half that observed in children of the same age, larger food particles at the swallowing threshold, and chewing sequences of approximately twice the duration. The very small convenience sample of eight children with DS limits the precision and generalizability of the findings and does not support the establishment of normative thresholds [46]. Ferrari et al. conducted detailed baseline and texture assessments in four children whose usual diet consisted of puréed foods. No components of chewing were observed at baseline, and the assessment protocol identified different starting points for teaching biting, chewing, and tongue lateralization. This case-based assessment is clinically informative but does not demonstrate that a specific intervention improves dietary intake or nutritional outcomes [45].

Studies based on caregiver reports and observational assessments further broaden this clinical picture. In a comparison of 96 young children with DS and 31 typically developing children, Arslan reported chewing difficulties in 39 children (40.6%), drooling in 30 (31.3%), and coughing during swallowing in 40 (41.7%), based on caregiver questionnaires and functional scales [56]. Anil et al. compared 17 children aged 2–7 years with 47 typically developing children and identified difficulties across all phases of swallowing, which were most pronounced with solid foods, together with poorer orosensorimotor performance [55]. Both studies support the need for multidimensional assessment; however, their small samples or substantial reliance on caregiver-reported measures limit their ability to estimate population-level rates.

### 5.2. Sensory Properties, Texture, and Eating Behavior

Food texture encompasses both mechanical and sensory demands. Hardness, brittleness, cohesiveness, particle size, graininess, moisture content, and mixed consistencies alter the force, timing, and bolus control required during eating. Oral sensory hyporesponsivity may manifest as mouth overstuffing, prolonged oral holding, or seeking intense sensory input, whereas hyperresponsivity may manifest as gagging, food refusal, or a strong preference for foods of a particular temperature or texture. These patterns should be described behaviorally, and the label “sensory” should not be used to bypass assessment for dysphagia, pain, or insufficient feeding skills [47,49,62].

A home-based study conducted in the United States included 111 children with DS aged 11–58 months and 107 typically developing children. The assessment combined parent-reported measures with coded video recordings of the children consuming standardized commercially available snack foods [62]. Children with DS more frequently moved away from the food and engaged in mouthing or sucking behaviors, while chewing or mashing less and consuming smaller amounts. Their behavior frequently resembled that of younger, typically developing children [48]. A subsequent analysis identified texture as the most influential sensory attribute: grainy or angular products were associated with coughing, choking, or gagging, while mouth overstuffing varied according to food texture and size [49]. Direct observation complements caregiver reports by documenting behavior during the feeding sessions, but it does not establish causal relationships or long-term outcomes. However, only a limited selection of snack foods was examined within a brief, structured context. Consequently, no conclusions can be drawn regarding habitual nutrient intake, swallowing safety, or long-term food preferences.

Parent-reported ease of eating also varies according to food texture, but these findings should not be interpreted as a universal hierarchy of texture difficulty [47,62]. A dissolvable solid may be manageable for one child but unsafe for another. Mixed consistencies may require rapid separation and coordination of their components; raw vegetables and fibrous meats may exceed the child’s chewing capacity, and smooth foods may be rejected because of oral aversion. A clinically useful nutritional assessment therefore classifies foods according to both their nutritional role and their sensory and mechanical properties. The intervention can then bridge the gap between an accepted food and a nutritionally valuable related food by modifying one property at a time, while the feeding therapist determines whether the child possesses the motor pattern required to manage it [48,49].

### 5.3. Food Selectivity and Neophobia

Food selectivity refers to a limited or rigid dietary repertoire, often related to food texture, taste, appearance, or method of preparation. Food neophobia refers to the rejection of unfamiliar foods, whereas food selectivity may involve the rejection of both familiar and unfamiliar foods [42,43]. These constructs are distinct from dysphagia, although they may coexist with sensory difficulties and underdeveloped feeding skills [7].

Food refusal describes a behavior but does not explain its underlying cause. It may represent a protective response when a child cannot safely process or swallow a food bolus [9,10,11,12,13,45,46], result from pain, reflux, constipation, or choking [51,53], or reflect a response to an unpleasant sensory stimulus and limited exposure to unfamiliar foods [63]. Before attributing refusal to noncompliance or sensory avoidance, swallowing safety, oral motor abilities, pain and other medical causes, and the appropriateness of the offered texture should be assessed [7,9,10,11,12,13].

Estimates of the prevalence of food selectivity and neophobia are limited by inconsistent definitions, measurement instruments, age ranges, and assessment methods [43,64]. Because of differences in participant age, sample characteristics, and the measurement instruments used, findings from studies specifically involving children with DS should be compared with caution [30,31].

De Almeida et al. [31] surveyed caregivers of 231 Brazilian children with DS aged 4–11 years. Based on caregiver responses to the Brazilian Children’s Food Neophobia Questionnaire, 41.1% were classified as having high food neophobia. Convenience recruitment through snowball sampling and the absence of objective dietary assessment limit generalizability [31]. In a Polish online survey of caregivers of 310 individuals with DS aged 4 months to 25 years and 10 months, 26.5% were classified as having moderate to very severe feeding difficulties and 41.3% as having high food neophobia. Higher neophobia was associated with lower caregiver-reported consumption of several food groups [30]. These questionnaire-based classifications should not be interpreted as clinical diagnoses or population prevalence estimates, and the mixed-age Polish sample does not provide a separate pediatric estimate. The related home-based studies described in Section 5.2 help distinguish rejection of an unfamiliar food from refusal of a familiar food whose texture exceeds the child’s sensory or motor abilities [48,49,62]. Analyses of the same dataset should be interpreted together rather than counted as independent replication. Their brief snack-tasting assessments do not establish habitual nutrient intake or swallowing safety. Caregiver questionnaires and direct observation provide complementary information about food refusal and mealtime interactions [18,19,20]. Family resources and food availability may also restrict the repertoire; these relationships are discussed in Section 5.4 [21,29].

The nutritionist should quantify the dietary repertoire rather than merely describe the child as a “picky eater”. Assessment should include regularly accepted foods and textures, the quantity and frequency of consumption, rigidity regarding preparation or brand, foods that have been lost from the repertoire over time, and the child’s response to the gradual introduction of new foods. The number of accepted foods should be interpreted alongside their nutritional value, as several nutritionally similar snack products do not constitute a diverse diet. Assessment of the dietary repertoire should therefore be combined with an analysis of energy, fluid, protein, fiber, and micronutrient intake, longitudinal growth monitoring, and targeted laboratory markers when clinically indicated [30,31,52,65].

Intervention should first address pain and safety barriers and ensure that food texture is appropriate for the child’s abilities. Once feeding safety has been established, the aim may be to gradually increase the acceptance of nutritionally important foods through calm prompting, repeated exposure, and preservation of the child’s autonomy [20,48,49]. Outcomes should be evaluated according to the amount actually consumed, dietary variety, adequacy of intake, meal duration and emotional tone, and feasibility for the family. The available evidence remains insufficient to recommend a single standardized protocol for treating food selectivity or neophobia specifically in children with DS [14,18,19,20,30,31,66,67].

### 5.4. Caregiver Practices, Mealtime Burden and Social Context

Feeding takes place within the relationship between the child and the person providing care. Caregivers must balance concerns about the child’s growth and development, fear of aspiration, time constraints, siblings’ food preferences, advice from different professionals, and the child’s autonomy. These concerns may lead to pressure to eat, distraction during meals, continuous grazing, preparation of a separate preferred meal following food refusal, or restriction of access to food because of concerns about obesity. Such responses may be understandable and, in some circumstances, necessary in the short term. However, they become problematic when they obscure the child’s hunger and satiety cues, perpetuate a restricted food repertoire, or make the feeding plan impractical for the family [18,19,66,67].

In an observational analysis of 212 video-recorded snack sessions involving 111 dyads of parents and children in which the child had DS, autonomy-supportive prompts were followed by consumption of the target food or completion of the task within 20 s in 49.3% of instances, compared with 24.2% following coercive or controlling prompts [20]. This measure of immediate success does not demonstrate a sustained improvement in dietary intake. Nevertheless, the findings support a low-pressure feeding approach based on modeling, structured choices, and clear instructions rather than increasing coercion. In a separate pilot study of 32 young children aged 1–3 years, caregiver-reported feeding difficulties were associated with observed food refusal and negative affect; however, the group as a whole did not perform more poorly than normative comparison samples [18].

Rogers et al. [19] compared 40 children with DS with 40 age- and sex-matched peers and identified a greater number of feeding problems among children with DS. These problems were associated with food avoidance behaviors, slower drinking, and a shorter duration of breastfeeding. Barreiro et al. [66] prospectively assessed 68 children aged 2–7 years and found that parental concerns and perceptions differed according to the child’s body mass index z-score. In the United Arab Emirates, Osaili et al. described feeding problems alongside restrictive feeding practices, pressure to eat, and a focus on body weight in a clinic- and school-based sample [67]. None of these study designs can determine whether caregiver feeding practices caused the child’s behavior or developed in response to it.

A qualitative study conducted in Rwanda involving 34 caregivers recruited from four hospitals described delayed texture progression, financial constraints, anatomical difficulties, and variable levels of family and professional support [21]. The children’s ages were not collected, and prevalence could not be estimated. Nevertheless, the study identified mechanisms that may be overlooked in clinical research conducted in high-income countries. In Croatia, dietary patterns among 104 children varied according to place of residence, maternal education, and household resources. Access to home-produced food did not automatically result in a healthier diet. Clinical advice to “offer more fish, nuts, and vegetables” is incomplete if it does not account for cost, availability, safe food textures, cooking skills, school meals, and the time required to feed the child [29]. Table 3 summarizes key publications on food texture, food neophobia, and caregiver feeding practices, including complementary analyses of eating behaviors, food sensory properties, and parental feeding practices from the same home-use study [20,48,49].

## 6. Medical Conditions Affecting Feeding and Nutritional Risk

### 6.1. Cardiorespiratory and Airway Conditions Affecting Feeding

Congenital heart disease may increase energy requirements and the work of breathing while simultaneously reducing feeding endurance. Tachypnea, diaphoresis, frequent pauses, and early fatigue may shorten effective feeding time, whereas fluid restriction increases the need for the carefully calculated energy and nutrient density of each feed [4,24,69,70]. In hemodynamically stable infants awaiting surgery, enteral nutrition may be provided with appropriate monitoring. When feeding volume is limited, fortified human milk, energy- or protein-enriched infant formula, and partial or full enteral nutrition support may be required [69,70]. Energy requirements may be elevated in unrepaired congenital heart disease, remain difficult to predict during the perioperative period, and approach those of healthy peers following surgery. The nutrition prescription should therefore be reassessed after hemodynamic improvement to ensure that a previously necessary energy-dense regimen does not become excessive [69,71]. Concentrating infant formulas increases nutrient delivery per unit volume but also changes osmolality and renal solute load. These preparation-related effects are described in pediatric renal nutrition guidance, which emphasizes individualized recipes and caregiver education [72]. This source provides indirect guidance on feed preparation rather than evidence of DS-specific nutritional requirements.

Upper airway obstruction, sleep-disordered breathing, laryngomalacia, tracheobronchomalacia, and recurrent respiratory infections are common and interrelated contributors to respiratory morbidity in children with DS [73,74]. In cohorts of children with chronic respiratory symptoms who underwent bronchoscopic evaluation, multiple airway abnormalities were frequently identified, and dysphagia was associated with more pronounced markers of airway inflammation [75]. Dysphagia and frequently silent aspiration may present clinically as chronic cough, wheezing, or recurrent lower respiratory tract infections; therefore, respiratory symptoms should not automatically be attributed to the syndrome itself or to infection. However, coughing during feeding does not in itself confirm aspiration. It is important to determine whether symptoms are temporally associated with feeding and whether an instrumental swallowing assessment would alter the prescribed food or fluid consistency, flow rate, or route of feeding [9,10,11,12,13,74].

### 6.2. Gastrointestinal, Endocrine, and Oral Health Conditions

Pain and gastrointestinal symptoms may precipitate or perpetuate food avoidance. Gastroesophageal reflux, vomiting, abdominal bloating, and constipation may reduce appetite or cause the child to associate mealtimes with discomfort [51,53]. Assessment should distinguish inadequate fluid and fiber intake and restrictions related to safe food textures from adverse medication effects, structural disease, celiac disease, and thyroid dysfunction. When fluid or fiber intake is below age-appropriate levels, gradual normalization is warranted. A review of pediatric studies found inconsistent evidence for the effectiveness of fiber supplementation in functional constipation [76]. The type and form of fiber-rich foods should be adapted to the child’s chewing and swallowing abilities and total fluid intake. These dietary modifications should be incorporated into a broader, individualized management plan for functional constipation in accordance with current pediatric clinical guidelines [77]. Probiotics are not routinely recommended because the available evidence does not demonstrate consistent efficacy in treating functional constipation in children [78].

Celiac disease requires particular attention because growth faltering, iron deficiency, persistent constipation, abdominal pain or bloating, and changes in behavior or feeding may be nonspecific [79,80,81,82]. Health supervision guidelines for children with DS recommend active surveillance for these manifestations and serological testing when indicated [3]. A retrospective study from a single specialized center involving 1317 children with DS found that routine screening increased case detection and reduced diagnostic delay. This finding supports consideration of periodic screening but does not, by itself, establish a universal testing schedule [81]. Diagnostic evaluation should be performed while the child is consuming gluten and should include measurement of serum IgA tissue transglutaminase antibodies (tTG-IgA) and total IgA, with an appropriate alternative diagnostic algorithm in cases of IgA deficiency [83]. A strict gluten-free diet is an evidence-based treatment only after the diagnosis has been confirmed [83,84]. Because this diet may further restrict the intake of fiber, iron, and certain vitamins and increase the practical and financial burden on the family, specialist dietary education is required [85]. Empirical gluten exclusion before completion of the diagnostic evaluation may compromise diagnostic accuracy and unnecessarily narrow the child’s dietary repertoire.

Thyroid dysfunction may contribute to altered growth, fatigue, constipation, and changes in body weight and may develop despite normal newborn screening results. Guidelines for children with DS recommend measuring thyroid-stimulating hormone at 6 and 12 months of age and annually thereafter, or earlier if symptoms develop [3]. Prospective monitoring of infants with DS during the first year of life further supports the need for repeated biochemical surveillance [86]. When treatment is indicated, dietary measures cannot substitute for levothyroxine. Dietetic management should focus on consistent medication administration in relation to meals and appropriate temporal separation from iron and calcium supplements or enteral nutrition, in accordance with the prescriber’s instructions and follow-up laboratory results, because these exposures may reduce levothyroxine absorption [87]. Iodine and seaweed supplements should not be used empirically: both inadequate and excessive iodine intake may disrupt thyroid function, and the iodine content of seaweed is highly variable [88].

Delayed tooth eruption, malocclusion, dental caries, gingival or periodontal inflammation, and oral pain may alter the food textures that a child is able or willing to chew [3,89,90,91]. Systematic reviews indicate a higher prevalence of certain forms of malocclusion and more pronounced periodontal inflammation in children with DS, whereas comparative data on dental caries are heterogeneous and do not support the assumption that all children with DS are at greater risk than their peers. A sudden onset of food selectivity, unilateral chewing or food pocketing on one side, and refusal of previously accepted textures should prompt an oral and dental examination rather than being automatically attributed to sensory difficulties. Regular preventive dental care is therefore an integral component of feeding assessment [3]. Frequent consumption of snacks or beverages high in free sugars and the repeated use of sugar-containing medications may increase the risk of dental caries [92].

## 7. Nutritional Status and Dietary Risks

### 7.1. Growth Faltering, Undernutrition, and Dehydration

Children with DS have a characteristic growth pattern marked by lower birth weight, slower linear growth, and shorter final adult height. DS-specific growth charts allow body weight, length or height, head circumference, and BMI to be monitored and enable comparison with children of the same age and sex who also have DS. These charts serve as reference and screening tools, but they do not determine whether growth is optimal or identify the cause of any deviation [93,94]. AAP guidance recommends their use throughout childhood, together with the general CDC BMI charts after 10 years of age, which are more sensitive for detecting excess adiposity [32,94]. Growth should therefore be assessed using serial, standardized measurements and interpreted in the context of preterm birth, congenital heart disease, thyroid dysfunction, celiac disease, feeding difficulties, and pubertal development [94].

Low body weight or growth faltering may result from inadequate intake, increased requirements, nutrient losses, malabsorption, or an underlying disease. Anthropometric indicators alone cannot distinguish among these mechanisms [95,96]. Assessment should therefore include actual food and fluid intake, evaluated using a 24-h dietary recall and/or a detailed multiday dietary record. The offered and consumed volumes, spillage, meal duration, recipes, formula concentration, meal fortification, and safe food textures should be documented [7,97]. This can help determine whether a prolonged meal provides little actual intake or whether texture-modified foods have insufficient energy and nutrient density. A low BMI may indicate undernutrition, but it does not confirm an isolated protein deficiency. Albumin should likewise not be used to confirm or exclude protein-energy malnutrition because its concentration primarily reflects inflammation and disease severity [96,98].

A child with dysphagia may develop dehydration if the prescribed consistency, method of serving, or poor acceptance of a beverage reduces the volume actually consumed below requirements. Thickening itself does not reduce the intestinal absorption of water when the child consumes the same volume. Actual intake and beverage tolerance should therefore be monitored rather than assuming that a thickener automatically reduces water availability [58]. Hydration assessment should integrate information on intake and losses, changes in body weight, and multiple clinical signs, with laboratory testing when indicated. No single clinical sign is sufficiently reliable on its own [99]. Total water intake includes water from beverages, infant formula, and foods. After changing a thickener or formula concentration, the amounts of water and nutrients actually consumed should be recalculated. Concentrating infant formula increases its potential renal solute load; therefore, the preparation should not be altered by empirically adding powder [100].

### 7.2. Dietary Patterns and Micronutrient Status

Available studies indicate opportunities to improve the diets of children with DS but do not define a single diet specific to the syndrome. In a Polish clinical sample of 39 children aged 9–17 years, 15% had obesity and 23% were overweight. Only a small proportion of children met the recommended consumption frequencies for vegetables, whole grains, plain dairy products, nuts, seeds, and oily fish. Children with overweight or obesity had higher food enjoyment scores, although the overall food interest score did not differ significantly [28]. In another cross-sectional study involving 143 adolescents with DS and 100 matched controls, the median Healthy Eating Index-2015 score was higher in the DS group (52.7 vs. 45.1), although diet quality was rated as low in both groups. Within the DS group, the total index score was not significantly associated with the measured cardiometabolic factors [27].

Survey studies provide information on food consumption frequency and dietary restrictions but do not allow reliable assessment of individual nutrient intake. In a Polish online survey, caregivers reported on 195 individuals with DS aged 0.5–30 years, including eight adults; 26.2% reported gluten avoidance and 28.7% reported lactose avoidance. Vegetables and fruit were most commonly consumed once daily, whereas 20% of participants did not consume dairy products. Because the clinical indications for the elimination diets and age-specific nutrient intakes were not objectively verified, these findings represent caregiver reports rather than confirmed diagnoses or nutrient deficiencies [52]. In a Croatian study of 104 children, more than 60% had never consumed whole grain bread, more than half did not consume fish, nuts, or seeds, and more than 30% of children aged 1–15 years were overweight. The cross-sectional design likewise precludes causal conclusions [29].

Feeding behavior is also associated with dietary diversity. In a Polish online study involving 310 caregivers, a high risk of food neophobia was associated with less frequent consumption of vegetables, fish, legumes, nuts, and whole grain bread [30]. A US online survey of 44 families of young children with DS found that the families implemented fewer than 15% of the assessed recommended feeding practices and were less likely to serve vegetables or model healthy eating. However, the cross-sectional design, reliance on parental reporting, and overrepresentation of families with higher educational attainment and income limit the generalizability of these findings. The result should not be interpreted as the percentage of children whose diets were nutritionally adequate. Collectively, these findings support the concurrent monitoring of dietary diversity, feeding behavior, and anthropometric status but do not establish what proportion of biochemical abnormalities can be attributed to feeding difficulties [68].

Micronutrient status in children and adolescents with DS has previously been examined in a systematic review and meta-analysis [39], while an earlier meta-analysis considered individuals with DS across age groups [101]. The present section draws on these findings to discuss their relevance to nutritional assessment in children with feeding difficulties. It does not update the pooled estimates. Differences in micronutrient concentrations between groups do not, by themselves, establish deficiency in an individual child, indicate the population prevalence of clinically diagnosed deficiency, or demonstrate that feeding difficulties caused the observed biochemical differences [39,101]. Iron status requires targeted monitoring because macrocytosis may reduce the sensitivity of conventional erythrocyte indices. DS-specific studies have shown that assessments incorporating ferritin, transferrin saturation, or other appropriate indicators detect iron deficiency more reliably than hemoglobin or mean corpuscular volume alone. Health supervision guidelines therefore recommend annual hematologic and iron status assessments beginning at one year of age [3,65,102,103].

A small observational study reported lower 25-hydroxyvitamin D concentrations in children and adolescents with DS, particularly among those with obesity or autoimmune disease [104]. These findings do not establish an appropriate supplementation dose or demonstrate a benefit of universal high-dose supplementation. The 2024 Endocrine Society guideline addresses vitamin D supplementation for disease prevention in individuals without established indications for testing or treatment; it should not be interpreted as a DS-specific protocol for treating confirmed deficiency [105]. The same principle applies to other micronutrients. Dietary and clinical risks should be assessed individually, an appropriate biomarker should be selected, and the response to treatment should be evaluated. Commercial hair mineral analysis is not sufficiently reliable for diagnosing an individual nutritional deficiency or determining the need for supplementation [106]. Direct evidence on dietary intake and nutritional status is summarized in Table 4.

### 7.3. Feeding Difficulties and Excess Adiposity

A restricted dietary repertoire does not necessarily imply inadequate energy intake. A child may obtain sufficient or excessive energy from a narrow selection of accepted soft or energy-dense foods while consuming limited dietary variety and inadequate amounts of particular nutrients. This provides a plausible explanation for the coexistence of feeding difficulties, excess body weight, and nutritional risk, but the complete pathway has not been established longitudinally. In the caregiver survey of 310 individuals with DS, reported feeding difficulties and food neophobia were not significantly associated with reported weight status [30]. Dietary studies and clinical recommendations therefore support assessing feeding function, food variety, energy intake, and nutrient adequacy as related but distinct aspects of care [27,28,29,30,108]. Lower resting energy expenditure may contribute to differences in energy requirements, although its role in the development of excess adiposity remains uncertain. In a three-year prospective study involving 28 children with DS and 35 of their siblings, resting energy expenditure adjusted for lean mass was lower in the DS group. No association with subsequent gains in fat mass was detected [109]. Given the small DS sample, this null finding should be interpreted cautiously and does not rule out a contribution of lower resting energy expenditure to later adiposity. Energy requirements should be assessed individually and reviewed alongside changes in growth and body weight. BMI is a useful screening measure but is not a direct measure of adiposity. Among individuals with DS aged 10–20 years, the general CDC BMI charts were more sensitive than DS-specific charts for identifying excess fat mass measured by dual-energy X-ray absorptiometry [32]. In another study, adolescents with DS had lower fat mass and lean mass at the same BMI z-score but a higher prevalence of dyslipidemia and prediabetes than comparison participants without DS [36]. Assessment should therefore extend beyond a single BMI category and include the longitudinal BMI trajectory, blood pressure, clinical comorbidities, and laboratory assessment of lipids, glucose metabolism, and liver function when indicated by age and degree of adiposity [110].

Evidence regarding treatment remains limited. A 2025 systematic review included 24 distinct studies with a total of 1171 participants, 393 of whom had DS. The studies differed substantially in intervention components, duration, sample size, and representation of participants with DS, precluding the identification of a single optimal intervention [111]. In a secondary analysis of an 18-month randomized trial, 53 participants with DS achieved mean body weight changes of −0.5%, −0.7%, and −4.0% in the conventional face-to-face intervention, conventional remote intervention, and enhanced remote Stop Light Diet groups, respectively. Because this was a secondary analysis and the individual groups were small, these results do not establish the superiority of any one intervention specifically for individuals with DS [107].

Treatment should be family-centered, multicomponent, and adapted to the child’s developmental and functional abilities. The plan should preserve accepted nutrient-dense foods and safe textures while modifying energy intake through adjustments to portion sizes, sugar-sweetened or other energy-dense beverages, and unstructured snacking. Dietary variety should be expanded gradually without abruptly removing most of the child’s accepted foods. Depending on age, growth trajectory, degree of adiposity, and comorbidities, the initial goal may be to slow excessive weight gain, maintain body weight, or achieve gradual weight loss. Available evidence supports caregiver involvement and an adapted multicomponent intervention rather than a restrictive “DS-specific diet” [108,110].

## 8. Nutritional and Feeding Interventions

Clinical management should address the causes and consequences of the child’s feeding difficulty. The recommendations below distinguish DS-specific findings from indirect pediatric evidence and clinical practice recommendations. Evidence origin is separate from strength of support: a DS-specific study may be small or uncontrolled, whereas general pediatric guidance may provide a well-established standard of care. Where outcomes have not been evaluated in children with DS, efficacy in this population remains uncertain. During infancy, general lactation guidance and DS-specific literature support early, skilled, and noncoercive breastfeeding support, with close monitoring of milk transfer, intake, growth, and feeding safety [22,23,26,112]. Supportive measures may include appropriate positioning, shorter and more frequent breastfeeds, breast compression, and timely expression of milk when direct milk transfer is inadequate. Supplemental expressed human milk, infant formula, fortification, or enteral nutrition may be introduced when necessary, while seeking to preserve the family’s lactation goals without compromising the infant’s safety or growth [22,23,26].

Clinical guidance, informed by findings from DS-specific dysphagia cohorts, supports targeting the identified physiological impairment when dysphagia is confirmed or considered highly likely. Possible modifications include reducing flow rate, adjusting feeding pace and position, using adapted feeding equipment, offering smaller boluses, thickening liquids, modifying food texture, and providing partial or complete nonoral feeding. The aim is to use the least restrictive strategy that simultaneously ensures safety and adequate intake, with regular reassessment because respiratory status, anatomy, and feeding skills change as the child grows [9,10,12,16].

General pediatric guidance supports enteral nutrition when nutritional requirements cannot be met safely or effectively through oral feeding [113,114]. Its use is not necessarily permanent. DS-specific observational evidence comes from a recent cohort in which approximately half of the participants discontinued gastrostomy feeding during follow-up [115]. The plan should clearly specify whether the enteral route is intended to provide complete nutrition, partial supplementation, or hydration and, when safe, should preserve opportunities for oral experiences and feeding skill development [114]. Energy, protein, fluid, fiber, and micronutrient intake should be reassessed periodically in children receiving tube feeding. When administered at the prescribed volume, a nutritionally complete commercial enteral formula may meet reference nutrient requirements. However, low prescribed volumes and inappropriate use of modular supplements may result in an imbalanced nutrient intake [113]. Guidance on blenderized tube feeding is drawn from the broader pediatric literature and emphasizes assessment of nutritional composition, microbiological safety, viscosity, feeding tube diameter, medication compatibility, and caregiver burden [116,117,118]. Because no comparative studies of commercial formula feeding and blenderized tube feeding specific to children with DS were identified, the choice of regimen should be individualized rather than based on an assumed universal advantage of either approach.

DS-specific intervention evidence for oral motor, sensory, and behavioral feeding therapy remains limited [40,41]. Franceschetti et al. evaluated Global Intensive Feeding Therapy in 20 children with DS with a mean age of 4.86 years. Following 15 sessions delivered over five days, improvements were observed in chewing performance and feeding measures based on IDDSI, both immediately after the program and at the one-month follow-up. However, the study lacked a control group, all participants screened negative for dysphagia risk, and nutrient intake, growth, and respiratory outcomes were not assessed [40]. In a 2025 randomized study, 38 children aged 3–10 years were assigned either to an eight-week home-based occupational therapy program or to a control group without intervention. Improvements were reported in sensory functioning, food selectivity, food refusal, and feeding skills; however, the conclusions are limited by the small sample, short follow-up, reliance on questionnaire-based outcomes, and absence of data on nutrient intake, growth, or feeding safety [41]. These studies provide preliminary evidence of improvement in feeding function, including chewing, food acceptance, and feeding skill measures [40,41]. They do not establish improved nutritional adequacy or feeding safety. Intervention outcomes should therefore be considered separately as feeding-function outcomes (chewing, texture acceptance, and feeding behavior), nutritional outcomes (energy and nutrient intake, dietary diversity, growth, body composition, and biochemical status), and safety outcomes (aspiration, choking, respiratory events, and swallowing physiology). Improvement in one domain should not be assumed to imply improvement in another.

Recommendations for behavioral and responsive feeding combine clinical practice principles with observational findings in children with DS; their long-term nutritional effects remain uncertain [20]. Behavioral and responsive feeding strategies should be introduced only after pain, medical causes of food refusal, and swallowing safety have been assessed. A predictable schedule of meals and snacks, caregiver modeling, small portions intended for learning, limited structured choices, neutral exposure, and praise for participation may reduce mealtime conflict. Observational evidence favors autonomy-supportive prompts over coercive prompting, although no longitudinal evidence demonstrates that this approach improves nutritional status over the long term [20]. Therapeutic goals should therefore be specific, such as accepting a defined texture, chewing effectively, reducing meal duration, incorporating a nutritionally important food, or increasing independent feeding, with the same observable outcome measure applied at baseline and during follow-up.

Once a safe texture has been established, general pediatric nutrition principles can guide adjustments to energy and nutrient density according to the child’s intake, growth, and nutritional needs. Cereal-based foods, purées, and soft foods may be fortified with human milk, infant formula, dairy products, eggs, meat, pulses, oils, or smooth nut and seed butters when allergen exposure and texture are considered safe. An increase in energy intake must be accompanied by adequate protein and micronutrient intake, because fortification with fat or carbohydrates alone may increase body weight while leaving iron, zinc, or protein intake inadequate.

Expert recommendations emphasize adapting efforts to improve dietary quality to the child’s feeding abilities and patterns of food acceptance [108,119]. If vegetables, fish, or nuts are rejected because of their texture or odor or because chewing skills are insufficiently developed, the aim should not be to insist on a single form of the food but to identify a safe and nutritionally comparable alternative [29,119]. In children with excess adiposity, priority should be given to structuring meals and snacks, making water the default beverage when it can be consumed safely, reducing sugar-sweetened beverages and energy-dense foods of low nutritional value, and adjusting portion sizes without removing accepted nutrient-dense foods. Physical activity and sleep management complement dietary intervention but must be adapted to the child’s motor abilities. Small studies of swimming and active video games suggest possible benefits for physical fitness or body composition. However, these activities do not constitute treatment for feeding difficulties and do not justify using exercise to compensate for food intake [120,121,122,123]. Weight management programs for individuals with intellectual disabilities further emphasize the importance of frequent contact, accessible materials, self-monitoring, caregiver involvement, and non-stigmatizing goals, although long-term outcomes specific to people with DS remain uncertain [107,111,124].

A documented nutritional deficiency should be treated in accordance with appropriate pediatric guidelines. However, a diagnosis of DS alone is not an indication for broad-spectrum or high-dose supplementation. Heterogeneous differences at the group level in zinc, selenium, and other micronutrient concentrations do not demonstrate that prophylactic supplementation improves feeding, growth, immune function, or neurodevelopment, while the concurrent use of multiple products may result in excessive intake and interactions [39,101,125]. DS-specific randomized trials have likewise failed to demonstrate the anticipated neurodevelopmental benefits: antioxidant and folinic acid supplementation did not improve developmental outcomes in a trial involving 156 infants [126], while epigallocatechin gallate did not improve cognitive outcomes or adaptive functioning in the PERSEUS trial [127]. A gluten-free diet is indicated in confirmed celiac disease, and allergen exclusion is appropriate in children with a diagnosed food allergy. Other restrictive diets are not supported for DS itself, and every clinically necessary exclusion should be accompanied by a plan to replace the displaced energy, protein, fiber, and micronutrients [48,83,84].

## 9. Strength of Evidence, Clinical Implications, and Research Priorities

The clinical contribution of this synthesis is to interpret feeding function and nutritional status together. Guideline-supported monitoring and clinically indicated assessment provide a basis for care, while the effects of individual treatments require separate evaluation [3,7]. A child’s accepted textures, volume consumed, nutrient density, growth trajectory, and family circumstances may identify different priorities even when BMI is similar. This framework organizes assessment; it does not demonstrate that a particular multidisciplinary package is superior to alternative care. Thyroid surveillance and other condition-specific monitoring remain guided by DS health-supervision recommendations [3,128], while lactation support draws on both DS-specific studies and guidance for hypotonic infants [22,23,24,25,26,112]. Evidence supporting specific therapeutic approaches is less certain. Preliminary studies suggest that intensive multidisciplinary feeding therapy and home-based programs targeting sensory processing and feeding skill development may be beneficial [40,41]. Observational findings support teaching caregivers strategies that promote children’s autonomy during mealtimes [20], while tailored multicomponent weight management interventions may benefit selected children and adolescents [107,111,124]. However, because of small sample sizes, short follow-up periods, and intervention heterogeneity, the children most likely to benefit, optimal treatment intensity and duration, required professional composition of the team, and strategies for maintaining improvements have not yet been clearly established [14,40,41,111]. Outcomes important to families, such as mealtime participation, treatment acceptability, caregiver burden, and quality of life, are also rarely assessed [14,40,111]. These interventions should therefore be implemented with explicit communication of the underlying uncertainty and systematic monitoring of predefined outcomes.

An absence of evidence should be distinguished from evidence of no effect. Failure to measure nutritional or respiratory outcomes in a feeding therapy study cannot establish that the intervention has no such benefit [40,41]; conversely, improved feeding scores cannot establish that benefit. Negative findings from trials of particular supplements should be interpreted in relation to the tested product, dose, population, and outcome [126,127]. Energy-requirement estimates also require individual review because a small study reporting reasonable agreement at group level does not establish a universally applicable DS-specific prediction equation [129]. The uncertainty surrounding many recommendations reflects the fragmentation of the evidence according to age, clinical setting, and assessment methods [14,30,55]. Studies of dysphagia are predominantly retrospective and susceptible to referral bias [9,10,11,12,13]. Feeding assessments frequently rely on caregiver reports, and some questionnaires have been applied outside their validated age ranges [18,19,30]. Dietary studies are primarily cross-sectional and rarely relate food and nutrient intake to objectively assessed feeding function [27,28,29,30,52,68]. Intervention studies are generally small, short in duration, and heterogeneous [14,40,41,111]. Children receiving enteral nutrition and those with severe developmental or medical comorbidities are underrepresented in studies of complementary feeding and feeding interventions [14,40,41], although clinical cohorts indicate that respiratory disease, aspiration, and substantial functional limitations may markedly affect feeding modality and nutritional risk [3,9,57].

Geographical and socioeconomic concentration also limits the applicability of the evidence. Findings from specialist services and relatively advantaged families may not transfer directly to settings with limited access to feeding assessment, affordable nutrient-dense foods, or suitable school meals. Evidence from Rwanda and Croatia illustrates the relevance of family resources, place of residence, and access to support [21,29]. Clinical recommendations should therefore be adapted to local food availability, cultural practices, household resources, and the child’s feeding abilities. Advice that is nutritionally appropriate may still be impractical or unsafe if these conditions are not considered.

Future prospective multicenter studies should follow children from birth and assess them at predefined developmental time points. Milk transfer and swallowing should be evaluated alongside texture progression, feeding skill development, dietary diversity, nutrient intake, growth, body composition, and respiratory outcomes. Such an approach would help clarify the temporal relationships between feeding difficulties, dietary intake, and growth. Instrumental swallowing studies should clearly describe referral criteria, the textures and viscosities tested, oral and pharyngeal physiology, penetration and aspiration, and responses to feeding modifications. Feeding assessments should use age-appropriate validated instruments and distinguish dysphagia, delayed feeding skills, sensory avoidance, food neophobia, and other domains of pediatric feeding disorder.

Intervention studies should link changes in feeding safety, skills, and behavior to meaningful nutritional and clinical outcomes. They require multicenter samples, allocation concealment in randomized trials, blinded outcome assessment, sufficiently long follow-up, and reporting of adverse events, intervention acceptability, and caregiver burden. A minimum outcome set should include the safety and efficiency of oral intake, respiratory events, meal duration, texture and dietary diversity, adequacy of nutrient intake, growth or body composition, participation, and quality of life. An improved questionnaire score may represent a meaningful functional outcome but does not by itself establish nutritional or safety benefits. Economic evaluations are also needed because the costs of professional support, food, thickeners, and equipment, as well as caregiver time, may influence the accessibility and sustainability of interventions.

The external validity of future evidence will depend on the inclusion of children from countries with different income levels, rural communities, culturally diverse dietary settings, and families with limited access to specialist care. Co-designing research with children and caregivers may facilitate the selection of relevant outcomes and reduce participation burden. Multidisciplinary registries are useful for monitoring rare complications and long-term outcomes but cannot replace comparative intervention studies. Dietitians should be included in research teams to ensure that changes in feeding function are related to actual energy and nutrient intake and to measurable changes in nutritional status.

## 10. Conclusions

Feeding difficulties in children and adolescents with DS arise from interactions among medical conditions, swallowing function, feeding skills, food acceptance, and family circumstances. Their nutritional implications may change across development, from inadequate milk and fluid intake in infancy to restricted dietary variety and excess adiposity later in childhood. Feeding function and nutritional status should therefore be assessed together. Adequate or excessive body weight does not exclude poor dietary quality, insufficient intake of particular nutrients, or unsafe or inefficient feeding.

This review brings these dimensions together within a developmental framework to inform individualized, multidisciplinary care. However, the proposed relationships should not be interpreted as a proven causal sequence. Evidence for specific feeding and nutritional interventions remains limited, and improvements in chewing, food acceptance, or feeding scores do not by themselves establish better nutritional status or swallowing safety. Recommendations should be adapted to the child’s abilities, medical needs, and family resources. Longitudinal and intervention studies are needed to determine whether improvements in feeding function lead to sustained gains in dietary adequacy, growth, safety, and participation in family meals.

## Figures and Tables

**Table 1:** Definitions of key terms used in this review.

Term	Definition and Use in This Review
**Feeding difficulty**	A broad descriptive term for problems with obtaining, processing, accepting, or consuming food or liquids. It does not identify a specific diagnosis or underlying cause.
**Pediatric feeding disorder**	Impaired oral intake that is not age-appropriate and is associated with medical, nutritional, feeding skill, and/or psychosocial dysfunction [7].
**Dysphagia**	Difficulty swallowing that may affect the safety or efficiency of oral intake. Dysphagia is one possible component of a feeding difficulty, but the terms are not interchangeable [7,9,10,11,12,13].
**Food refusal**	Rejection of offered food or liquids. It describes an observable behavior rather than its cause, which may involve discomfort, swallowing difficulty, limited feeding skills, or food-related preferences [7,18].
**Food selectivity**	A limited or rigid repertoire of accepted foods, often influenced by texture, taste, appearance, or preparation. It may involve rejection of both familiar and unfamiliar foods [42,43].
**Food neophobia**	Reluctance to try or accept unfamiliar foods. It is distinct from rejection of familiar foods and does not, by itself, indicate a swallowing impairment [42,43].

**Table 2:** Key pediatric studies of feeding skill, dysphagia and aspiration in DS.

Study	Design and Sample	Assessment	Principal Finding	Critical Limitation
Stanley et al., 2019 [9]	Retrospective specialty clinic cohort; 174 infants, 0–6 months.	Clinical screening; VFSS when concern identified	100/174 were referred for VFSS; 96/174 had oral and/or pharyngeal dysphagia. 69/174 required altered consistency or nonoral feeds.	Single-center; testing triggered by concern; not a population prevalence estimate
Jackson et al., 2019 [11]	Retrospective referred cohort; 111 infants; 247 VFSS and 14 FEES examinations.	Instrumental swallowing evaluation	Deep penetration or aspiration occurred in 31.9% of infants < 6 months and 51.3% at 6–12 months.	Multiple examinations per child; referral and clinical-selection bias
Narawane et al., 2020 [10]	Retrospective VFSS cohort; 127 referred infants.	VFSS oral and pharyngeal-phase findings	89.8% had at least one oral-phase abnormality and 72.4% had pharyngeal dysphagia. Abnormal sucking and bolus control were frequent.	Highly selected referred sample; chart review dependence
Jackson et al., 2016 [12]	Retrospective initial VFSS cohort; 158 children; mean age 2.10 years.	VFSS and clinical records	Pharyngeal dysphagia in 56.3%. 61 aspirated and 90.2% of aspiration was silent. Oral findings did not predict pharyngeal dysphagia.	Tertiary referral cohort; retrospective; broad age range
O’Neill and Richter, 2013 [13]	Tertiary chart review; 201 children with complete records.	VFSS-confirmed pharyngeal dysphagia and follow-up	116/201 had pharyngeal dysphagia. Resolution was documented in 20 during available follow-up.	Older, referral-enriched study; non-standardized follow-up
Anil et al., 2019 [55]	Comparative study; 17 children with DS aged 2–7 years and 47 typically developing peers.	Questionnaire and orosensorimotor evaluation	Difficulty affected all swallowing phases and was greatest for solids. Orosensorimotor performance was poorer.	Very small DS sample (n = 17); limited precision and generalizability
Serel Arslan, 2023 [56]	Comparative study; 96 toddlers with DS and 31 typically developing toddlers.	Caregiver-reported PEDIEAT-10, TFSIS and functional items	Chewing disorder, drooling and coughing during swallowing were frequently reported. Scores and caregiver burden were higher in DS.	Caregiver report; no instrumental confirmation of aspiration
Tanrıverdi et al., 2025 [57]	Cross-sectional study; 335 children aged 4–12 years.	FOIS, STEP and clinical observation	25.9% classified as nonoral feeders and 33.7% at aspiration risk. Younger age associated with higher risk.	Aspiration risk was screened, not instrumentally confirmed. Likely selected case mix

Abbreviations: FEES, fiberoptic endoscopic evaluation of swallowing; FOIS, Functional Oral Intake Scale; STEP, Screening Tool of Feeding Problems; VFSS, videofluoroscopic swallow study.

**Table 3:** Key Studies of Factors Shaping Food Acceptance and Dietary Intake in Children with DS.

Study	Design and Sample	Exposure/Outcome	Nutrition-Relevant Result	Critical Limitation
Hielscher et al., 2023 [14]	Scoping review; 18 studies; 1276 children	Complementary feeding timing and early eating experience	Evidence suggested delayed solids/texture progression and motor, sensory and caregiver contributors.	Only two intervention studies; no longitudinal study; Western and clinic samples predominated.
Ross et al., 2022 [48]	Home-use comparison; 111 children with DS aged 11–58 months and 107 typically developing peers	Researcher-coded eating behavior and snack consumption from parent-recorded videos	More distancing and mouth/suck behavior, less chewing/munching and lower intake in DS; behavior resembled younger peers.	Short experimental snack context; not habitual nutrient intake.
Ross et al., 2024 [49]	Secondary sensory analysis of the home-use study; 111 children with DS aged 11–58 months	Sensory profiling of test foods and researcher-coded eating behavior	Texture was the dominant property; grainy or angular products were associated with coughing, choking, or gagging; size/texture related to overstuffing.	Commercial test foods; cannot diagnose aspiration or establish causality.
Surette et al., 2023 [20]	Observational analysis of parental feeding practices within the home-use study; 111 parent–child dyads	Researcher-coded parental prompts and immediate child responses	Autonomy-supportive prompts were successful in 49.3% versus 24.2% for coercive-controlling prompts.	Immediate response only; socially advantaged US sample; no longitudinal diet outcome.
Rogers et al., 2022 [19]	Cross-sectional matched comparison; 40 DS and 40 typically developing children, 6 months–5 years	Parent-reported feeding problems, eating behavior and practices	DS group had more feeding problems; associations involved food avoidance, slow drinking and breastfeeding duration.	Small DS sample (n = 40); caregiver-reported measures; cross-sectional associations do not establish direction or causality.
Białek-Dratwa et al., 2025 [30]	Online caregiver survey concerning 310 individuals with DS aged 4 months to 25 years and 10 months	Caregiver-completed MCH-FS and FNSC; caregiver-reported food frequency and anthropometry	26.5% moderate-to-very-high feeding difficulty; 41.3% high neophobia; high neophobia aligned with lower intake of several nutrient-dense groups.	Mixed pediatric and adult sample; MCH-FS used beyond its validated age range; caregiver-reported data.
De Almeida et al., 2025 [31]	Convenience online survey; 231 Brazilian children aged 4–11 years	Validated Brazilian food neophobia questionnaire	41.1% classified with high neophobia; school appeared favorable for new fruit exposure.	Chain recruitment and caregiver perception; no dietary or biochemical validation.
Clarke et al., 2026 [68]	Online survey; 44 DS, 42 autistic and 98 comparison families	Recommended family nutrition practices	DS families met <15% of practices measured and reported less vegetable serving and healthy eating modeling.	Small DS sample (44 families); overrepresentation of families with higher education and income; practice scores do not measure nutrient adequacy.
Rugema et al., 2026 [21]	Qualitative exploratory study; 34 caregivers from four Rwandan hospitals	Focus groups and interviews	Delayed progression, affordability, anatomy and variable support shaped feeding decisions.	Convenience sample; no child ages; qualitative findings do not provide population prevalence estimates.

**Table 4:** Direct evidence on dietary intake, nutritional status and downstream risk.

Study	Design and Sample	Measures	Key Result	Interpretive Caution
Wernio et al., 2022 [28]	Cross-sectional; 39 children aged 9 to <18 years	FFQ, CEBQ, anthropometry, DXA, lipids and oxidative markers	15% had obesity and 23% overweight; several nutrient-dense food groups were underconsumed; adiposity related to lipid/oxidative markers.	Small sample; multiple comparisons; cross-sectional associations.
Anand et al., 2023 [27]	Cross-sectional secondary analysis; 143 DS and 100 matched adolescents	Three 24-h recalls, HEI-2015 and cardiometabolic markers	Median HEI-2015 was 52.7 in DS versus 45.1 in controls, but low in both; no significant within-DS association with cardiometabolic measures.	Recall error; selected hospital cohort; no feeding phenotype measure.
Białek-Dratwa et al., 2023 [52]	Online caregiver survey concerning 195 individuals with DS aged 0.5–30 years	Caregiver-reported food frequency and dietary exclusions	26.2% reported gluten and 28.7% lactose restriction; vegetables and fruit were most often consumed once daily.	Mixed pediatric and adult sample; caregiver-reported data; clinical indications for dietary exclusions not verified; nutrient intake not quantified.
Ergović Ravančić et al., 2025 [29]	Cross-sectional online Croatian survey; 104 children	Food frequency, anthropometry and socioeconomic context	>60% never consumed whole grain bread and >50% avoided fish, nuts or seeds; over 30% aged 1–15 were overweight.	Caregiver report and self-selection; no biochemical outcomes.
Osaili et al., 2019 [67]	Cross-sectional UAE clinic/school sample	Anthropometry and parent–child feeding questionnaires	Stunting and excess weight coexisted with feeding problems and counterproductive parent–child practices.	Selected setting; cross-sectional and questionnaire-based.
Barišić et al., 2023 [39]	Systematic review and meta-analysis across ages	Circulating micronutrient concentrations	A distinct average micronutrient profile was reported, including trace element differences.	Age, assay, inflammation and supplement heterogeneity; group means are not individual diagnoses.
Hart et al., 2020 [65]	Multicenter pediatric DS study	CBC, ferritin and additional iron indices	Expanded hematological and iron markers improved detection compared with limited blood-count interpretation.	Diagnostic performance depends on biomarker definitions and inflammatory context.
Ptomey et al., 2022 [107]	Post hoc DS subgroup (n = 53) of 18-month randomized weight trial	Weight, BMI and intervention adherence	At 18 months, mean DS weight change ranged from −0.5% to −4.0% across three multicomponent arms.	Small exploratory subgroup; attrition; no isolated feeding intervention

## Data Availability

No new data were created or analyzed in this study. Data sharing is not applicable to this article.

## Abbreviations

The following abbreviations are used in this manuscript:

AAP	American Academy of Pediatrics
BMI	Body mass index
CBC	Complete blood count
CDC	Centers for Disease Control and Prevention
CEBQ	Children’s Eating Behavior Questionnaire
DS	Down syndrome
DXA	Dual-energy X-ray absorptiometry
FEES	Fiberoptic endoscopic evaluation of swallowing
FFQ	Food frequency questionnaire
FNSC	Food Neophobia Scale for Children
FOIS	Functional Oral Intake Scale
HEI-2015	Healthy Eating Index–2015
IDDSI	International Dysphagia Diet Standardisation Initiative
IgA	Immunoglobulin A
MCH-FS	Montreal Children’s Hospital Feeding Scale
MEDLINE	Medical Literature Analysis and Retrieval System Online
PEDIEAT-10	Pediatric version of the Eating Assessment Tool-10
STEP	Screening Tool of Feeding Problems
T-FS-IS	Turkish version of the Feeding/Swallowing Impact Survey
UAE	United Arab Emirates
US	United States
VFSS	Videofluoroscopic swallow study
